# Exploring the Anti-*Burkholderia cepacia* Complex Activity of Essential Oils: A Preliminary Analysis

**DOI:** 10.1155/2014/573518

**Published:** 2014-02-19

**Authors:** Isabel Maida, Antonella Lo Nostro, Giovanna Pesavento, Martina Barnabei, Carmela Calonico, Elena Perrin, Carolina Chiellini, Marco Fondi, Alessio Mengoni, Valentina Maggini, Alfredo Vannacci, Eugenia Gallo, Anna Rita Bilia, Guido Flamini, Luigi Gori, Fabio Firenzuoli, Renato Fani

**Affiliations:** ^1^Department of Biology, University of Florence, Via Madonna del Piano 6, Sesto Fiorentino, 50019 Florence, Italy; ^2^Department of Health Sciences, University of Florence, Viale G. B. Morgagni 48, 50134 Florence, Italy; ^3^Center for Integrative Medicine, Careggi University Hospital, University of Florence, 50139 Florence, Italy; ^4^Department of Chemistry Ugo Schiff, University of Florence, Via della Lastruccia 3-13, Sesto Fiorentino, 50019 Florence, Italy; ^5^Department of Pharmacy, University of Pisa, Via Bonanno 33, 56126 Pisa, Italy; ^6^Laboratory of Microbial and Molecular Evolution, Department of Biology, University of Florence, Via Madonna del Piano 6, Sesto Fiorentino, 50019 Florence, Italy

## Abstract

In this work we have checked the ability of the essential oils extracted from six different medicinal plants (*Eugenia caryophyllata*, *Origanum vulgare*, *Rosmarinus officinalis*, *Lavandula officinalis*, *Melaleuca alternifolia*, and *Thymus vulgaris*) to inhibit the growth of 18 bacterial type strains belonging to the 18 known species of the *Burkholderia cepacia* complex (Bcc). These bacteria are opportunistic human pathogens that can cause severe infection in immunocompromised patients, especially those affected by cystic fibrosis (CF), and are often resistant to multiple antibiotics. The analysis of the aromatograms produced by the six oils revealed that, in spite of their different chemical composition, all of them were able to contrast the growth of Bcc members. However, three of them (i.e., *Eugenia caryophyllata*, *Origanum vulgare*, and *Thymus vulgaris*) were particularly active versus the Bcc strains, including those exhibiting a high degree or resistance to ciprofloxacin, one of the most used antibiotics to treat Bcc infections. These three oils are also active toward both environmental and clinical strains (isolated from CF patients), suggesting that they might be used in the future to fight *B. cepacia* complex infections.

## 1. Introduction

Essential oils (EOs) consist of a complex blend of volatile and fragrant substances typically synthesized by all plant organs as secondary metabolites and extracted by water or steam distillation, solvent extraction, expression under pressure, supercritical fluid, and subcritical water extractions [1]. EOs include two biosynthetically related groups, mainly terpenes and terpenoids and, secondarily, aromatic and aliphatic constituents, all of them characterized by low molecular weight. Biological properties of EOs terpenoids are not well elucidated but a function of protecting plants against predators and microbial pathogens is postulated and they could be important in the interaction of plants with other organisms (e.g., attraction of pollinators). The same plant species can produce different EOs chemotypes (i.e., chemical components). For example, *Thymus vulgaris*, morphologically identical species with a stable karyotype, consist of seven different chemotypes depending on whether the dominant component of the essential oil is thymol, carvacrol, linalool, geraniol, sabinene hydrate, *α*-terpineol, or eucalyptol.

In recent years, the emergence of bacterial resistance against multiple antibiotics has accelerated dramatically. The quinolones/fluoroquinolones, azole, and polyene classes of antimicrobials often are the last resort to treat infections; hence the chances of acquiring resistance against these antimicrobials are higher [2]. EOs and other plant extracts possess antibacterial, antifungal, and antiviral properties and have been screened worldwide as potential sources of novel antimicrobial compounds [3]. Thus EOs and their constituents can hopefully be considered in the future for more clinical evaluations and possible applications and as adjuvants to current medications [4]. The antimicrobial properties of EOs have been reported in several studies. High antimicrobial activity of *Thymus* and *Origanum* species has been attributed to their phenolic components such as thymol and carvacrol and those of *Eugenia caryophyllus*, *Syzygium aromaticum*, and *Ocimum basilicum* to eugenol [1]. In fact thyme and oregano EOs can inhibit some pathogenic bacterial strains such as *Escherichia coli*, *Salmonella enteritidis*, *Salmonella cholerasuis*, and *Salmonella typhimurium,* with the inhibition directly correlated to carvacrol and thymol [5]. The mechanisms by which essential oils can inhibit microorganisms involve different modes of action and in part may be due to their hydrophobicity. As a result, they get partitioned into the lipid bilayer of the cell membrane, rendering it more permeable, leading to leakage of vital cell contents [6]. There are fewer reports on the mechanisms of action of EOs combination or their purified components on microorganisms. They include the sequential inhibition of a common biochemical pathway, inhibition of protective enzymes, and use of cell wall active agents to enhance the uptake of other antimicrobials. The capacity of hydrocarbons to interact with cell membrane facilitates the penetration of carvacrol into the cell. In many cases the activity results from the complex interaction between the different classes of compounds such as phenols, aldehydes, ketones, alcohols, esters, ethers, or hydrocarbons found in EOs [1]. It is likely that it will be more difficult for bacteria to develop resistance to the multicomponent EOs than to common antibiotics that are often composed of only a single molecular entity [3]. For example the multicomponent nature of tea tree oil could reduce the potential for resistance to occur spontaneously, since multiple simultaneous mutations may be required to overcome all of the antimicrobial actions of each of the components. This means that numerous targets would have to adapt to overcome the effects of the oil [7].

Clinical studies with EOs are scarce. Topical use is the most promising strategy at the moment, for both skin and mucous membranes. Some hope exists for inhalation uses, but clinical evaluation is needed. There is little information regarding safety in relation to oral administration of EOs, so an increase in the knowledge about pharmacokinetics, pharmacodynamics, and the potential toxicity of EOs administered by this route is required [3].

Particularly interesting from this viewpoint is the possibility to treat infections of cystic fibrosis (CF) patients. One of the most important opportunistic CF pathogens is represented by bacteria belonging to the *Burkholderia cepacia* complex (Bcc) belonging to the very heterogeneous genus *Burkholderia*, which currently comprises more than seventy species, isolated from wide range of niches. Many members of the genus can cause infection in plants, animals, and humans, and most studies have thus focused on these pathogenic species due to their clinical importance [8]. However, recently, an increasing number of *Burkholderia *species associated with plants or with the environment and able to fix nitrogen, to nodulate legume or to promote plant growth, were described [8]. Among the pathogenic species, the Bcc bacteria, a group of genetically distinct but phenotypically similar bacteria that up to now comprises 18 closely related bacterial species [9, 10], have become known as opportunistic pathogens in humans. Although they are not considered important pathogens for the normal human population, some of them are considered serious threats for specific patient groups such as CF patients [11]. CF is the most fatal genetic disease of Caucasians [9], and the main cause of morbidity and mortality in patients is chronic lung infection involving different species of bacteria (mainly *Pseudomonas aeruginosa*), fungi, and viruses [12]. Regarding Bcc species, the prevalence (2009 and 2010) of chronic infection is reported to vary between 0 and 12% of the CF population attending various CF centres [13]. Although it is not high compared to other CF pathogens, Bcc infections correlate with poorer prognosis, longer hospital stays, and an increased risk of death [14].

One of the reasons for the high rate of mortality in infections caused by Bcc species is their high resistance to antibiotics: they are intrinsically resistant to many antibiotics and can develop *in vivo* resistance to essentially all classes of antimicrobial drugs [14, 15]. This high antibiotics resistance is the result of mechanisms specific for certain classes of antibiotics and of an intrinsic resistance, characteristic of all Gram-negative bacteria, due to the cooperation between the outer membrane barrier and the expression of efflux systems [14, 16]. Between multidrug efflux systems, the intrinsic drug resistance of Gram-negative bacteria is mainly attributable to RND (resistance-nodulation-cell division protein family) type drug exporters [17]. The presence and distribution of these kinds of proteins in some available *Burkholderia *genomes are known [18, 19], and some of these systems have also been experimentally characterized [20–23].

New antimicrobial agents are always needed to counteract the Bcc resistant mutants that continue to be selected by current therapeutic regimens. Bacterial resistance often results in treatment failure that causes severe aftermath especially in critically ill patients [24]. Inappropriate or unnecessary antibiotic prescriptions, the excessive use of antibiotics in the agricultural and livestock industries, and the lack of patient adherence to full antibiotic regimens, all of which select resistant bacteria, appear to be the key contributors to the emergence of antibiotic resistance. Resistant bacteria may also spread and become broader infection-control problems, not only within healthcare institutions but within communities as well. For this reason there is a pressing need to develop new antibacterial therapies not only against Bcc bacteria but also against other different human pathogens [25]. In this context one of the most important approaches is represented by the search of new natural drugs from “unusual” sources; particularly interesting might be the essential oils since they are multi-component and, in principle, the probability of bacteria to develop resistance to this mixture of substances might be much lesser than to a single molecule.

Therefore, the aim of this work was to explore the antimicrobial activity of six different essential oils versus a panel of Bcc bacteria, some of which exhibiting multiresistance to different drugs and with either clinical or environmental source, in order to check the possibility of using essential oils to fight Bcc infections in CF patients.

## 2. Materials and Methods

### 2.1. Bacterial Strains and Growth Conditions

The bacterial strains used in this work are listed in Table 1. They were grown either on Tryptone Soya Agar (TSA, Oxoid S.p.A., Strada Rivoltana, 20090 Rodano, MI, Italy) medium at 37°C for two days or in liquid Tryptone Soya Broth (TSB, Oxoid S.p.A., Strada Rivoltana, 20090 Rodano, MI, Italy) medium at 37°C with shaking.

### 2.2. Aromatograms

#### 2.2.1. Preparation of Microbial Suspensions and Media

Each bacterial strain was grown at 37°C in liquid medium (TSB) with shaking; the growth was checked at regular time intervals (as spectrophotometric reading at OD_600_) until the end of the growth exponential phase was reached. Serial dilutions 1 : 10 to 10^−5^ of each bacterial suspension were plated on TSA Petri dishes in order to count the microorganisms and verify that the number of bacteria in the samples was appropriate to the performance of the tests.

TSA, used to perform the agar diffusion assays, was enriched with a suitable volume of Dimethylsulphoxide (DMSO, Carlo Erba Reagenti S.p.a., Strada Rivoltana km 6/7, 20090 Rodano, MI, Italy), sterilized by filtration through filters with a pore diameter of 0.22 *μ*m (Sartorius Italy Srl, Viale A. Casati 4, 20835 Muggiò, MB, Italy), thus obtaining 0.5% (v/v) solutions identified by the abbreviations of DTSA. The addition of DMSO, an aprotic organic solvent belonging to the category of sulfoxides, had the purpose of facilitating the solubilisation of essential oils in the aqueous medium represented by the culture media.

#### 2.2.2. Preparation of Dilutions of Essential Oils

The essential oils used in this study (*Eugenia caryophyllata*, *Origanum vulgare*, *Rosmarinus officinalis, Lavandula hybrida, Melaleuca alternifolia* and *Thymus vulgaris*) were all extracted by steam distillation method, and purchased from the same retailer (Prodotti Phitocosmetici Dott. Vannucci di Vannucci Daniela e C. Sas, Via la Cartaia Vecchia 3, 59021 Vaiano (PO), Italy). All EOs and EOs dilutions were stored at 4°C before use.

#### 2.2.3. Agar Disk Diffusion Assay

*Burkholderia* cell suspensions were streaked on DTSA Petri dishes. Sterile filter paper disks (Oxoid SpA. Strada Rivoltana, 20090 Rodano, MI, Italy) of 6 mm diameter were soaked with 10 *μ*L of each not diluted EO, and placed on the surface of the dishes. In addition, positive and negative controls were applied to the surface of agar plates; they were, respectively, the antibiotic ciprofloxacin (3 *μ*g/10 *μ*L) (Oxoid S.p.A. Strada Rivoltana, 20090 Rodano, MI, Italy) and a solution of DMSO 0.5% in sterile deionised water. The plates were incubated at 37 ± 1°C for 48 h aerobically. After incubation, the diameter of the inhibition zones was measured in millimeters, including the diameter of disk. The sensitivity to the EOs was classified by the diameter of the inhibition zones as follows: *not sensitive* for total diameter smaller than 8 mm, *Sensitive* for total diameter 9–14 mm, *very sensitive* for total diameter 15–19 mm, and *extremely sensitive* for total diameter larger than 20 mm [26]. Each assay was performed in triplicate on three separate experimental runs.

### 2.3. Determination of Essential Oil Composition

Gas cromatographic (GC) analyses were accomplished with an HP-5890 series II instrument equipped with a HP-5 capillary column (30 *μ*m × 0.25 mm, 0.25 *μ*m film thickness), working with the following temperature program: 60°C for 10 min, ramp of 5°C/min to 220°C; injector and detector temperatures, 250°C; carrier gas, nitrogen (2 mL/min); detector, dual flame ionization detection (FID); split ratio, 1 : 30; injection, 0.5 *μ*L. The identification of the components was performed, for both columns, by comparison of their retention times with those of pure authentic samples and by means of their linear retention indices (LRI) relative to the series of *n*-hydrocarbons. Gas chromatography-electron impact mass spectrometry (GC-EIMS) analyses were performed with a Varian CP 3800 gas chromatograph (Varian, Inc. Palo Alto, CA) equipped with a DB-5 capillary column (Agilent Technologies Hewlett-Packard, Waldbronn, Germany; 30 m × 0.25 mm, coating thickness 0.25 mm) and a Varian Saturn 2000 ion trap mass detector. Analytical conditions were as follows: injector and transfer line temperature at 250 and 240°C, respectively, oven temperature being programmed from 60 to 240°C at 3°C/min, carrier gas, helium at 1 mL/min, splitless injector. Identification of the constituents was based on comparison of the retention times with those of the authentic samples, comparing their LRI relative to the series of *n*-hydrocarbons and on computer matching against commercial and homemade library mass spectra built from pure substances and components of known samples and MS literature data [27–32]. Moreover, the molecular weights of all the identified substances were confirmed by gas chromatography-chemical ionization mass spectrometry (GC-CIMS), using methanol as chemical ionization gas.

### 2.4. Statistical Analyses

Inhibition zones in Bcc strains from the different EOs were analyzed by using principal component analysis as implemented in PAST software [33]. Kruskal-Wallis test with Bonferroni error protection was applied for comparing the overall inhibition zones from the different EOs by using the Analyse-it software (Analyse-it Software, Ltd.).

## 3. Results and Discussion

### 3.1. Composition of Essential Oils

Essential oils are very complex natural mixtures, which can contain about 20–60 components at quite different concentrations. They are characterized by two or three major components at fairly high concentrations (20–70%) compared to other components present in trace amounts. Terpenoids (mainly monoterpenoids and sesquiterpenoids) generally represent the principal constituents but some essential oils are characterised by the presence of aromatic (phenylpropanoids) and aliphatic constituents, all characterized by low molecular weight.

The tested essential oils were commercial samples and analysed by GC using as detector a dual FID and electron impact mass spectrometry. Constituents were identified by comparison of their retention times of both columns with those of pure authentic samples and by means of their linear retention indices (LRI) relative to the series of *n*-hydrocarbons and MS data from homemade library mass spectra and literature.

Almost 100% of the volatiles of oregano essential oil were identified, being 77.2% of oxygenated monoterpenes, principally represented by carvacrol representing 71.8% of the total essential oil; 19.2% of constituents were represented by monoterpene hydrocarbons, principally *p*-cymene; 2.9% were sesquiterpenes hydrocarbons, and 0.6% were oxygenated sesquiterpenes.

Also in the case of rosemary essential oil the identified volatiles were 99.9% and major constituents were represented by oxygenated monoterpenes (64.6%) being the main volatile 1,8-cineole (43.9%). Monoterpene hydrocarbons were 25.9%, principally *α*-pinene. Sesquiterpene hydrocarbons were 9.1% and oxygenated sesquiterpenes were only 0.3%.

Total identified constituents of thyme oil were 99.5%. These volatiles were characterized by 53.7% of monoterpene hydrocarbons being 47.9% *p*-cymene and oxygenated monoterpenes 45.6%, principally thymol (43.1%). Only 0.2% of the volatiles were sesquiterpenes hydrocarbons.

About 98% of constituents of clove oil were identified and the main metabolite was eugenol (85%), a typical phenylpropanoid, while 11.2% of the constituents were recognised as sesquiterpene hydrocarbons being *β*-caryophyllene the main molecule (9%).

Approximately all (99.1%) of the constituents of *M. alternifolia* were identified; principal compounds were oxygenated monoterpenes being 4-terpineol the principal one (39.9%). The rest of the oil was mainly represented by monoterpene hydrocarbons (41.4%) being *γ*-terpinene (14.4%) and *α*-terpinene (8.8%) the principal molecules.

### 3.2. Antimicrobial Activity of the Essential Oils against *Burkholderia cepacia* Complex (Bcc) Strains

The antimicrobial activity of the six different EOs (*E. caryophyllata *(Ec), *O. vulgare *(Ov), *R. officinalis *(Ro)*, L. hybrida *(Lh)*, M. alternifolia *(Ma), and *T. vulgaris *(Tv)) was checked versusthe 18 Bcc type strains listed in Table 1 and representative of the 18 known Bcc species; this panel comprises strains of either clinical or environmental origin.

**Figure 1:**
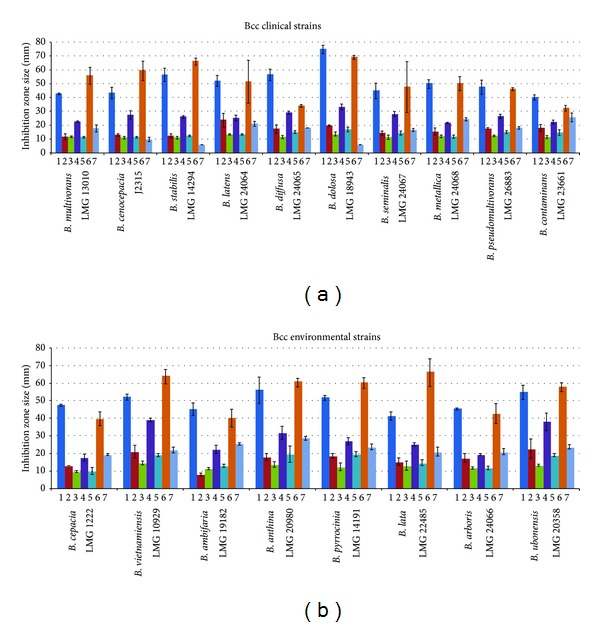
Inhibitory power of essential oils. Results for the agar diffusion assay performed on the 18 Bcc type strains are presented. Each bar of the histogram represents the mean of the inhibitory zone obtained for each of the EOs analyzed. In the graphics are reported the standard deviations for every arithmetic average obtained: (1) *Thymus vulgaris*, (2) *Rosmarinus officinalis*, (3) *Lavandula hybrida*, (4) *Eugenia caryophyllata*, (5) *Melaleuca alternifolia*, (6) *Origanum vulgare*, and (7) *Ciprofloxacin. *

Data obtained are reported in Figure 1 and showed the following.

- All the 18 bacterial strains, from both clinical and environmental origin, exhibited, although at a different extent, sensitivity to each of the six EOs tested.
- According to Ponce et al. [26], three essential oils, that is, Ec, Tv, and Ov, exhibited a very high inhibitory power *versus* all the Bcc strains tested. Indeed, all of them were *extremely sensitive* to these three EOs.
- Quite interestingly, these three EOs gave an inhibitory halo much larger than that produced by ciprofloxacin, suggesting that they are more active than this antibiotic.
- The other three EOs (Ro, Lh, and Ma) exhibited a degree of inhibition of Bcc growth lower than that exhibited by the three EOs mentioned above; however, the inhibitory halos they produced were similar and in many cases larger than those exhibited by ciprofloxacin.
- Apparently, clinical and environmental strains did not exhibit a different sensitivity to a given EO (or to a set of EOs), but they were differently sensitive to ciprofloxacin (Table 1). Two of them, that is, LMG 14294 (*B. stabilis*) and LMG 18943 (*B. dolosa*), were resistant to the antibiotic and *B. cenocepacia* J2315, representing the model system for the study of Bcc infection in CF patients, exhibited a low sensitivity to ciprofloxacin. These three strains have a clinical origin. In spite of this, the same three strains were extremely sensitive to the three most active EOs.
- Environmental Bcc strains were much more sensitive to ciprofloxacin than their clinical counterparts.

**Figure 2:**
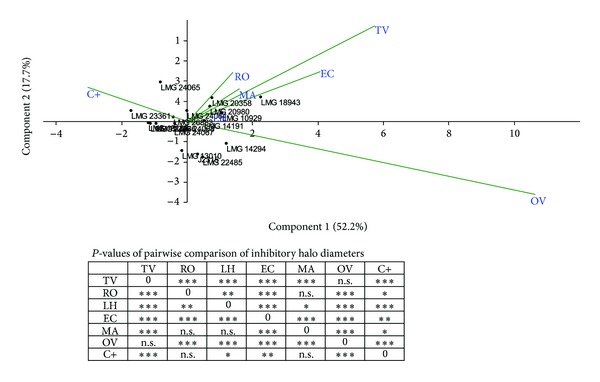
Differences in the patterns of inhibition of essential oils. Upper panel: principal component analysis biplot of inhibitory patterns 18 Bcc strains (centroids) treated with different EOs and ciprofloxacin (C+). The percentage of variance explained by the first two principal components is reported. Lower panel: *P* values of pairwise comparisons (Kruskal-Wallis test and Bonferroni error protection) between EOs and C+. n.s.: not significant; *P < 0.01; **P < 0.001; ***P < 0.0001.

The differential sensitivity to EOs and ciprofloxacin was confirmed by a principal component analysis (Figure 2). As shown in the biplot the vectors accounting for EOs are differentially oriented than those of ciprofloxacin (C+). Moreover, the vectors for Ov and Tv greatly contributed in the differential pattern of sensitivity, thus confirming that the most active essential oils were* T. vulgaris *and *O. vulgare. *Finally a pairwise comparison (Kruskal-Wallis test) of the patterns of inhibition of EOs and ciprofloxacin (Figure 2) showed that large differences between inhibitory halos of different EOs and ciprofloxacin are present, highlighting the observed (Table 1, Figure 1) differences in the inhibitory power of the six EOs.

## 4. Conclusions

In this work we have performed a preliminary analysis of the ability of six different essential oils to inhibit the growth of strains belonging to the *B. cepacia* complex, whose members are dangerous for CF patients; indeed they can cause severe infections in immune-compromised patients, such as those affected by cystic fibrosis. This idea relies on previous findings demonstrating that essential oils are able to inhibit the growth of some human pathogens, such as *E. coli*, *S. enteritidis*, *S. choleraesuis*, and *S. typhimurium* [5]. However, to the best of our knowledge, nothing are known on the ability of these mixtures of chemical compounds to inhibit the growth of Bcc members.

For this reason we selected six different essential oils (*E. caryophyllata*, *O. vulgare*, *R. officinalis, L. officinalis, M. alternifolia*, and *T. vulgaris*) that were tested *versus* a panel embedding the type strains of the known 18 Bcc species.

The composition of the six EOs was quite different but, in spite of this, all of them exhibited an inhibitory activity *versus* all the 18 Bcc strains, suggesting that one compound or (more likely) more than one compound (see below) present in each essential oil might interfere with the Bcc cell growth. However, the six essential oils showed a different inhibitory activity and according to Ponce et al. [26] they might be split into two different clusters; the first one includes *T. vulgaris, O. vulgare* and *E. caryophyllata*, whereas the other one embeds *R. officinalis, M. alternifolia* and *L. officinalis* (Table 2). Indeed, Bcc strains were extremely sensitive to the EOs belonging to the first group and just sensitive to the other three.

However, all of them are able to inhibit the growth of Bcc strains; particularly interesting and intriguing is the finding that the inhibitory halos produced by most of EOs are (much more) larger than those produced by ciprofloxacin, one of the antibiotics used in CF infections therapy. We are completely aware that the sensitivity to a given drug or to a complex mixture of antimicrobial compounds may strongly vary also between strains belonging to the same bacterial species. However, in our opinion, the preliminary data reported in this work are particularly encouraging, since they demonstrate that the use of essential oils might represent an alternative way to fight Bcc growth. It is also quite interesting that, in spite of the high number of experiments performed in this work, no Bcc mutant resistant to any of the essential oils tested was isolated (data not shown). This represents a very important finding, which strongly suggests that the ability of essential oils to inhibit the growth of Bcc cells might be very likely due to the simultaneous presence in the oil of different molecules (whose mechanism of action is still unknown) that might work in a synergistic fashion to antagonize the Bcc growth. In addition to this, in our opinion, these combinations of compounds should not act on a single target, but on different molecular targets within the Bcc cell. If this is so, the simultaneous block of the activity of different molecular targets should strongly decrease the probability of the appearance of a mutant able to resist the essential oils. If this scenario is correct, these data might pave the way to the use of essential oils to fight Bcc infection in CF patients.

## Figures and Tables

**Table 1:** List of bacterial strains used in this work and their sensitivity to the essential oils tested in this work.

*Burkholderia cepacia* complex strains									
Strain	Origin	Species	Sensitivity to						
			*Eugenia caryophyllata *	*Origanum vulgare *	*Rosmarinus officinalis *	*Lavandula hybrida *	*Melaleuca alternifolia *	*Thymus vulgaris *	Ciprofloxacin
LMG 13010	CF	*B. multivorans *	ES	ES	S	S	S	ES	VS
J2315	CF	*B. cenocepacia *	ES	ES	S	S	S	ES	S
LMG 14294	CF	*B. stabilis *	ES	ES	S	S	S	ES	NS
LMG 24064	CF	*B. latens *	ES	ES	ES	S	S	ES	ES
LMG 24065	CF	*B. diffusa *	ES	ES	VS	S	S	ES	VS
LMG 18943	CF	*B. dolosa *	ES	ES	VS	S	VS	ES	NS
LMG 24067	CF	*B. seminalis *	ES	ES	S	S	S	ES	VS
LMG 24068	CF	*B. metallica *	ES	ES	S	S	S	ES	ES
LMG 26883	CF	*B. pseudomultivorans *	ES	ES	VS	S	S	ES	VS
LMG 23361	AI	*B. contaminas *	ES	ES	VS	S	S	ES	ES
LMG 1222	Env	*B. cepacia *	VS	ES	S	S	S	ES	VS
LMG 10929	Env	*B. vietnamiensis *	ES	ES	ES	S	VS	ES	ES
LMG 19182	Env	*B. ambifaria *	ES	ES	NS	S	S	ES	ES
LMG 20980	Env	*B. anthina *	ES	ES	VS	S	ES	ES	ES
LMG 14191	Env	*B. pyrrocinia *	ES	ES	VS	S	ES	ES	ES
LMG 22485	Env	*B. lata *	ES	ES	S	S	S	ES	ES
LMG 24066	Env	*B. arboris *	ES	ES	VS	S	S	ES	ES
LMG 20358	Env	*B. ubonensis *	ES	ES	ES	S	VS	ES	ES

CF: strain isolated from cystic fibrosis patient; Env: environmental strain; AI: animal infection; NS, S, VS, and ES: not sensitive, sensitive, very sensitive, and extremely sensitive, respectively (according to Ponce et al., 2003) [26].

**Table 2:** Composition (%) and principal classes (%) of the six essential oils used in this work.

Constituents	LRI	Essential oil					
		*Lavandula hybrida *	*Eugenia caryophyllata *	*Melaleuca alternifolia *	*Origanum vulgare *	*Rosmarinus officinalis *	*Thymus vulgaris *
Tricyclene	928					0.2	tr
*α*-Thujene	933			0.6		tr	
*α*-Pinene	941	0.4	0.2	3.8	1.7	11.5	4.3
Camphene	955	0.3		tr	0.4	4.1	0.1
Thuja-2.4(10)-diene	959					tr	
Sabinene	977	0.1	tr	0.6			
*β*-Pinene	982	0.6	0.1	2.1	0.4	3.8	1.2
Myrcene	993	0.5		0.6	1.3	1.3	
*α*-Phellandrene	1006			0.4	tr	0.2	
1-Hexyl acetate	1010	0.1					
*δ*-3-Carene	1013	tr	tr				tr
1.4-Cineole	1018						0.1
*α*-Terpinene	1020		tr	8.8	0.8	0.4	
*p*-Cymene	1027	0.3	tr	3.7	11.6	1.9	47.9
Limonene	1032	0.7	0.1	2.0	1.1	1.8	0.2
1.8-Cineole	1034	6.9	tr	2.9	0.6	43.9	0.2
(*Z*)-*β*-Ocimene	1042	0.3					
*γ*-Terpinene	1063		tr	14.4	1.7	0.4	
*cis*-Sabinene hydrate	1070	0.1		tr		tr	
*cis*-Linalool oxide (furanoid)	1077	0.3					
Terpinolene	1090			4.4	0.2	0.3	
*trans*-Linalool oxide (furanoid)	1090	0.2					
1-Pentyl butyrate	1094				tr		
*trans*-Sabinene hydrate	1099			0.3			
Linalool	1101	27.1			1.8	0.9	1.2
1-Octenyl acetate	1112	0.4					
*exo*-Fenchol	1118			tr		tr	tr
*cis-p*-Menth-2-en-1-ol	1123			0.4			
Terpinen-1-ol	1135			0.2			
*trans*-Pinocarveol	1141					tr	
*trans-p*-Menth-2-en-1-ol	1142			0.4			
Camphor	1145	8.4			tr	11.3	
1-Hexyl isobutyrate	1152	0.2					
Isoborneol	1158				0.2		
*trans*-Pinocamphone	1162					tr	
Pinocarvone	1164					tr	
Borneol	1168	3.2			0.4	4.2	
Lavandulol	1171	0.6					
*cis*-Pinocamphone	1175					tr	
4-Terpineol	1178	3.9	tr	39.9	0.2	0.8	
*p*-Cymen-8-ol	1185			tr			
*α*-Terpineol	1190	1.7		4.2	0.4	2.6	0.6
1-Hexyl butyrate	1193	0.6					
*cis*-Piperitol	1195			tr			
Verbenone	1206					0.2	
*trans*-Piperitol	1207			0.2			
Nerol	1230	0.2					
1-Hexyl 2-methylbutyrate	1235	0.1					
1-Hexyl 3-methylbutyrate	1244	0.3					
Chavicol	1252		tr				
Linalyl acetate	1259	30.4					
*trans*-Ascaridolglycol	1268			0.2			
Isobornyl acetate	1287				0.2	0.7	
Lavandulyl acetate	1291	3.3					
Thymol	1292				1.6		43.1
Carvacrol	1301				71.8		0.4
1-Hexyl tiglate	1333	0.2					
*α*-Cubebene	1352		tr			tr	
Eugenol	1358		85.0				
Neryl acetate	1365	0.4					
*α*-Ylangene	1373					0.2	
*α*-Copaene	1377		0.2	tr	tr	0.6	
Geranyl acetate	1383	1.0					
*α*-Gurjunene	1410			0.5			
*β*-Caryophyllene	1419	2.2	9.0	0.5	2.7	5.1	0.2
Lavandulyl isobutyrate	1424	0.1					
*trans*-*α*-Bergamotene	1437	0.2					tr
*α*-Guaiene	1440			1.4		0.2	
*(Z)*-*β*-Farnesene	1444	0.2					
*α*-Humulene	1455	tr	1.4	0.1	0.2	0.5	tr
*(E)*-*β*-Farnesene	1459	1.1					
Alloaromadendrene	1461			0.6			
*γ*-Muurolene	1478					0.6	
Germacrene D	1482	0.3					
Valencene	1493			0.3			
Viridiflorene	1494			1.3		0.2	
Bicyclogermacrene	1496			0.7			
*α*-Muurolene	1499			0.2		0.2	
*β*-Bisabolene	1509	0.2				0.2	
Lavandulyl 2-methylbutyrate	1513	0.4					
*trans*-*γ*-Cadinene	1514	0.5				0.4	
*δ*-Cadinene	1524		0.6	1.8		0.9	
*trans*-Cadina-1(2).4-diene	1534			0.2			
Spathulenol	1577			0.2			
Caryophyllene oxide	1582	0.6	0.5		0.6	0.3	tr
Globulol	1584			0.5			
Guaiol	1597			0.2			
1-*epi*-Cubenol	1629			0.3			
T-Cadinol	1640	0.2					
Cubenol	1643			0.2			
*α*-Bisabolol	1684	0.4					
Monoterpene hydrocarbons		3.2	0.4	41.4	19.2	25.9	53.7
Oxygenated monoterpenes		88.2	0.0	48.7	77.2	64.6	45.6
Sesquiterpene hydrocarbons		4.7	11.2	7.6	2.9	9.1	0.2
Oxygenated sesquiterpenes		1.2	0.5	1.4	0.6	0.3	tr
Phenylpropanoids		—	85.0	—	—	—	—
Other derivatives		1.9	—	—	tr	—	—

Total identified		99.2	97.1	99.1	99.9	99.9	99.5

LRI: linear retention indices relative to the series of *n*-hydrocarbons; tr: traces.
